# RELATIONSHIPS AMONG ATTITUDES TOWARD DEMENTIA, QUALITY OF LIFE, AND MIDLIFE WOMEN’S SYMPTOMS IN FAMILY CAREGIVERS

**DOI:** 10.1093/geroni/igac059.3110

**Published:** 2022-12-20

**Authors:** Heejung Kim, Seo Yun Kim, Roger Yau, Glenna Brewster, Wonshik Chee, Eun Ok Im

**Affiliations:** Yonsei University, Seoul, Kyonggi-do, Republic of Korea; Emory University, Atlanta, Georgia, United States; Emory University, Atlanta, Georgia, United States; Emory University, Atlanta, Georgia, United States; Emory University, Atlanta, Georgia, United States; Emory University, Atlanta, Georgia, United States

## Abstract

Attitudes towards dementia and caregiving differ by family caregivers’ racial/ethnic backgrounds. However, there is a gap in the literature on midlife women family caregivers’ attitudes toward Alzheimer’s Disease (AD) and family caregiving. The study purposes were to (1) explore racial/ethnic variations in midlife women family caregivers’ attitudes toward AD and family caregiving and (2) examine the relationships among their attitudes towards dementia and caregiving, quality of life, and physical and psychological symptoms. This cross-sectional study was conducted through an online survey among 36 Whites, 41 African Americans, 40 Hispanics, and 55 Asians. The structured measures consisted of two types of attitudes (Attitude toward AD and Related Dementias Scale and Questions on Attitudes toward AD Caregiving), health-related quality of life (EQ-5D-5L), and multidimensional symptoms (Midlife Women’s Symptom Index). The data were analyzed using one-way ANOVA and multiple linear regression analyses with SPSS 26. Asian caregivers perceived the care recipients’ symptoms as more bothersome than White caregivers (p = .039). Asian caregivers reported lower levels of behavioral skills and shared responsibility compared with other racial/ethnic groups of caregivers (p < .01). African Americans showed more positive attitudes toward family caregiving compared with Hispanics and Asians (p = .001). The regression analyses indicated that more positive attitudes toward family caregiving were significantly related to a better quality of life and fewer symptoms (both physical and psychological symptoms; p < .05). Culturally tailored interventions that incorporate caregivers’ attitudes are needed to improve midlife women family caregivers’ quality of life and symptoms.

